# Hernia of the Jean Louis Petit triangle in the emergency department: a rare image

**DOI:** 10.11604/pamj.2020.37.166.10702

**Published:** 2020-10-15

**Authors:** Hassen Ben Ghezala, Najla Feriani

**Affiliations:** 1 Service Universitaire des Urgences et de Réanimation Médicale, Hôpital Régional de Zaghouan, Faculté de Médecine de Tunis, Tunis, Tunisie,; 2 Service Universitaire de Chirurgie Générale, Hôpital Régional de Zaghouan, Faculté de Médecine de Tunis, Tunis, Tunisie

**Keywords:** Lumbar hernia, lumbar triangle, surgery

## Image in medicine

Lumbar hernias are rare lesions. They are exceptionally observed and reported. They are in most of the cases secondary to trauma or previous surgery but primary lumbar hernias are rare. Computed tomography (CT) is a very useful tool for the diagnosis of lumbar hernia. It can delineate the neck of the hernia and hernial contents. All lumbar hernias must be treated with surgery with two possible surgical approaches: the anterior approach and the laparoscopic approach. Many techniques have been described, including primary repair, local tissue flaps and conventional mesh repair. Bowel resection may be required in cases with strangulation. We report in this work an exceptional case of a 43 year old man with a past recent medical history of lumbar trauma who attended the emergency room for a lumbar mass. It was a soft, non-tender, reducible swelling, measuring from five centimeters in diameter, sitting in the right lumbar region. Computed tomography was performed. The hernia was located in the superior triangle of Grynfelt-Lesshaft and contained large intestine of the right bowel. That is called hernia of the Jean Louis Petit triangle. The patient had open surgery (by lumbar incision). We reintroduced the intestine back into the peritoneal cavity. We excised the sac, and repaired the defect by using synthetic mesh placed below the muscular layers, using a tension-free technique. He was discharged at the fifth day from our hospital. He has been followed up for four years with no recurrence or postsurgical sequelae.

**Figure 1:**
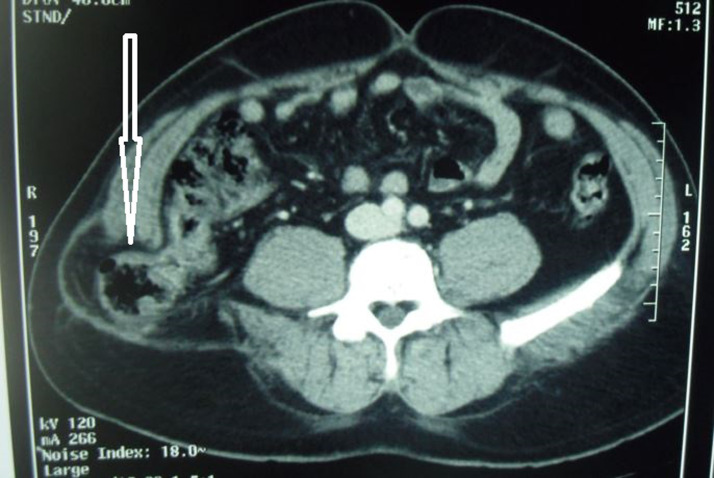
lumbar hernia containing large bowel through superior triangle (Grynfelt)

